# Dietary supplementation with yeast hydrolysate in pregnancy influences colostrum yield and gut microbiota of sows and piglets after birth

**DOI:** 10.1371/journal.pone.0197586

**Published:** 2018-05-24

**Authors:** Shah Hasan, Sami Junnikkala, Olli Peltoniemi, Lars Paulin, Annina Lyyski, Juhani Vuorenmaa, Claudio Oliviero

**Affiliations:** 1 Department of Production Animal Medicine, Faculty of Veterinary Medicine, University of Helsinki, Saarentaus, Finland; 2 Department of Veterinary Biosciences, Faculty of Veterinary Medicine, University of Helsinki, Helsinki, Finland; 3 Institute of Biotechnology, University of Helsinki, Helsinki, Finland; 4 Hankkija Oy, Peltokuumolantie 4, Hyvinkää, Finland; Wageningen University, NETHERLANDS

## Abstract

Dietary supplementation with yeast derivatives (YD) contributes to the health and physiology of sows and piglets, but few studies have focused on how it influences gut health and performance of sows and piglets. The goal was therefore to examine whether YD, based on brewer’s yeast hydrolysate added to pregnancy diet, would affect colostrum composition, yield (CY) and gut microbiota of sows and piglets. Sows were allocated to either a control diet (n = 19) or a control diet supplemented with 2g YD/kg (n = 18) during the pregnancy. Piglets suckling belonging to the control sows (n = 114) and supplemented sows (n = 108) were also included in the study. Gut microbiota populations of sows at farrowing and piglets at one and four weeks of age were assessed using 16S rRNA gene sequencing. Colostrum samples were examined for nutritional composition and immunoglobulin (Ig) content. All piglets were individually weighed at birth and 24 hours later in order to calculate CY, and later at four weeks to calculate average daily gain (ADG). Protein, lactose and dry matter content of colostrum did not significantly differ between the two groups, while sows fed YD had higher levels of fat in their colostrum (*P* < 0.05). Immunoglobulin A, IgM and IgG levels in colostrum did not differ between the two groups (*P* >0.05). Colostrum yield was lower in the control than that in YD group (3701g vs. 4581 g; *P* <0.05). Although the YD supplementation did not change fecal bacteria diversity in sow, more beneficial and fermentative bacteria (*Roseburia*, *Paraprevotella*, *Eubacterium*) were found in the YD fed group (*P* <0.01) while, some opportunistic pathogens, including *Proteobacteria*, especially the genera *Desulfovibrio*, *Escherichia/Shigella* and *Helicobacter*, were suppressed. Piglets at one week of age from sows fed YD had more beneficial microbial populations with significant diversity and fewer opportunistic pathogens. Additionally, we established a Pearson’s correlations between CY, colostrum components, piglet birth weight and fecal microbiota. Therefore, YD added to the sow diet during pregnancy increases colostrum availability and its energy content for neonate piglets, also promoting beneficial maternal microbial sources for neonate.

## Introduction

Yeast derivatives (YD) are widely used in animal nutrition as natural additives [1,2]. YD typically contains a complex of mannan oligosaccharides (MOS) and polysaccharides, glucomannoproteins, and betaglucans. They are commonly produced by enzymatic hydrolysis of different yeasts and contain mainly the insoluble cell wall fraction after separation of the soluble extract fraction. The most commonly used source is *Saccharomyces cerevisiae*, brewer’s yeast. The YD used in the study was derived from brewer’s yeast (*Saccharomyces cerevisiae*) by acid hydrolysis, containing both cell wall and extract fractions and a reasonably high level of soluble compounds such as oligosaccharides and peptides (detailed composition is shown in S2 Text). Studies indicated that dietary supplementation of YD improves sow performance, and significantly increases average daily gain of piglets and subsequent weaning weight [3,4]. Close and Taylor-Pickard [5] reported that dietary supplementation with YD improves colostrum production and quality and reduces piglet mortality. Colostrum yield is key factor in modern pig production where large litters are common. It was reported that approximately 30% of sows produce insufficient colostrum for their litter [6]. With constant increase in litter size, this is currently one of the major causes of neonatal piglet mortality in commercial pig production. Colostrum yield (CY) is associated with sow, piglet and environmental traits [7]. Therefore, feeding sows with alternative additives which may modulate their natural ability to improve CY is an important topic. However, to date, studies related to the YD supplementation of gestating sow diets and the effect on gut microbiota in sows and piglets are scarce. YD can bind and inhibit pathogen bacteria-like *Salmonella* spp., *Clostridium* spp. and *Escherichia coli*, therefore promoting growth of beneficial gut bacteria, better utilization of feed nutrients and reduced spread of pathogen to piglets [8–11]. YD have also been associated with positive immune stimulation by activation of alternative complement pathway, release of lysosomal enzymes, binding to the specific receptor of macrophages and cytokine in different animal species [12–15]. The first aim of this study was to determine the effects of YD inclusion in sows’ gestation diets on CY, colostrum immunoglobulins, nutritional composition and subsequent litter performance. The second aim was to investigate the influence of YD on the taxonomic profile of the hindgut microbiota of sows and piglets by the means of high-throughput sequencing analysis. Our hypothesis was that the inclusion of YD in a gestating diet would modify the hindgut microbiota of the sow and that the modifications could be associated with better colostrum yield, immunoglobulins content. We also hypothesized that piglets of the sows fed a YD diet would have better gut microbiota colonization, possibly contributing to better piglet performance.

## Materials and methods

The experimental protocol was approved by the National Animal Experiment Board in Finland (ESAVI, Regional State Administrative Agency for Southern Finland, permission ESAVI/333/04.10.03/2011). The experiment was carried out on a commercial pig farm in southern Finland from October 2015 to January 2016, the owner of the farm gave written permission to conduct the study in his farm. The experiment was repeated four times using four different batches of sows that farrowed during that time.

### Animals and experimental design

Parities of 37 multiparous sows (Yorkshire × Landrace of 1 to 8, 3.2 ± 0.3 parity) were balanced between the treatment groups. They were selected on the basis of start of farrowing by first come first sample–principle. Sow farrowed spontaneously and researcher (one of the authors) sampled all sows upon availability at farrowing. During gestation, sows were housed in groups of 15 to 20. The group housing rooms were equipped with individual feeding stalls and sows were fed a pregnancy diet (Tiineys Pekoni 87^®^, Table A in S1 Text). Sows were moved to a farrowing house ~1 week before the expected date of farrowing, kept in individual farrowing crates (200 cm × 80 cm), and given 3.0 kg of feed daily (Emakko Pekoni 106^®^, Table A in S1 Text). In both gestation and lactation, sows were fed the above control basal diets (CON diet; n = 19) or the same basal diets supplemented with 2g YD/kg feed YD (Progut^®^, Hankkija Oy/Suomen Rehu, Hyvinkää, Finland, product details in S2 Text) (YD diet; n = 18). Parturition was observed, with as little interference as possible in the farrowing process. The birth of the first piglet was considered to represent the beginning of parturition. During the first 24h piglets were allowed to consume only maternal colostrum and, piglets were provided with a milk supplement (Nuklospray Yoghurt^®^, Vilomix, Finland) after they had been weighted for colostrum intake analysis. Six piglets from each litter were selected and ear-tagged based on body weight at birth (BW_B)_ in a block of three categories, 2 piglets weight <1 kg, 2 piglets 1.4–1.8 kg and 2 piglets >1.8 kg, representing small, normal and large sized piglets, respectively. Cross-fostering was allowed only after 24 h weighing, it was only carried out within a treatment, except the six selected ear-tagged piglets. Litters were balanced according to the number of functional teats. Altogether 114 piglets belonged to sows fed with CON diet and 108 piglets belonged to sows fed with YD diet. Piglets were supplied with a dry creep feed after one week, in addition to maternal milk (Pikku-Pekoni Mini^®^, details creep feed composition in Table B in S1 Text). All the selected piglets were monitored until weaning at 4 weeks of age. Farrowing was not induced, and farrowing intervention was minimized to manual extraction of piglets when needed. No oxytocin was administered. No additional help or care was given to the piglets unless there was a risk of them becoming crushed by the sow.

### Parameters and measurements

All the piglets were weighed at birth, 24h after birth and at weaning (4 weeks of age). The CY was calculated as the sum of the individual piglets’ colostrum intake (CI) within a litter, as described by Devillers *et al*. [16] using the following variables: BW_B_ (kg), weight at 17 to 24 h of age (BW_24_, kg), duration of CI (t in min and 17 h ≤ t ≤ 25 h), and time between birth and first suckling (t_FS_, min). The regression equation was: CI = -217.4 + 0.217 × t + 1861019 × BW_24_/t + BW_B_ × (54.80-1861019/t) × (0.9985–3.7 × 10^−4^ × t_FS_ + 6.1 × 10^−7^ × t^2^_FS_). The tFS was estimated to be 35 min, which was based on our observations from previous studies [17] and was same as in a recent study [18]. An error of 15 min in t_FS_ will generate a 6g/kg BW_B_ miscalculation of CI for piglets or less than 2% error. Observed sow parameters were parity, gestation length, farrowing duration, and numbers of born and stillborn piglets. Observed piglet parameters were birth interval, pre-weaning mortality, BW_B_, BW_24_, body weight at weaning, and average daily gain (ADG). If needed, piglets were allowed to be cross-fostered after BW_24_ among the litters of the same treatment group, except for the six ear-tagged piglets that stayed with their original mother until weaning.

### Sampling colostrum, blood and feces

Twenty milliliters of colostrum were collected from each sow within the first two hours after the first piglet birth. Colostrum samples were collected from first three teats of same side of the anterior udder. Samples were subdivided and stored at –20°C until further analysis. Fresh fecal samples were individually collected from the rectum of sows using sterile 50 mL tubes (n = 18 CON; n = 18, YD). Piglet fecal samples were collected at 1 week (n = 32 CON; n = 46 YD) and 4 weeks of age (n = 32 CON; n = 50 YD) using sterile swabs and 5 ml Eppendorf tubes. After collection samples were kept in an icebox and transported immediately to the laboratory and stored at –80°C before total genomic DNA extraction. Sow blood samples were collected from the *vena saphena* at the beginning of farrowing using lithium heparin tubes and centrifuged at 1000 × g for 10 minutes, the plasma being separated and stored at -20°C for further analysis.

### Colostrum and blood sample analysis

The standardized and complete methods for colostrum nutritional composition (later on composition) are described in our previous study [17]. Concentration of Ig was quantified using swine IgG, IgM and IgA ELISA quantification kits (Bethyl Laboratories, Montgomery, Texas, USA). The intra- and inter-assay coefficients of variation were 4.8% and 6.7% respectively. The colostrum total solid (TS), fat, protein and lactose contents were analyzed using MilkoScan^TM^ FT+ (Foss, Hillerød, Denmark). Blood plasma progesterone was analyzed using radioiimunoassay (RIA) (Progesterone ImmuChem, ICN Pharmaceuticals, USA).

### Microbial characterization

Microbial genomic DNA was extracted from 250 mg of each fecal sample using a QIAamp DNA Stool DNA kit (Quagen, ct. no. 51504) according to the protocol described earlier [19]. The yield and purity of DNA extracts were quantified using a Nanodrop 2000 (Thermo Fisher Scientific). The 16S PCR amplification and sequencing was done as described in Pereira *et al*. [20] with modifications in primers. The 16s region amplified was V3-V4 and mixed primers 341F_1–4 (CCTACGGGNGGCWGCAG) and 785R_1–4 (GACTACHVGGGTATCTAATCC), with partial Illumina TruSeq adapter sequences added to the 5’ ends (details of adapter sequences present in S3 Text). The PCR amplification steps and MiSeq sequencing was done at the DNA Sequencing and Genomics Laboratory, Institute of Biotechnology, University of Helsinki, Finland, similarly as described by Pereira *et al*. [20]. Sequenced 16S rRNA gene amplicons were processed using MOTHUR software package (v 1.39.5) [21]. The two paired-end reads were joined and the sequences were demultiplexed and quality filtered with the removal of sequences containing bases <200 bp. Sequences were assigned to operational taxonomic units (OTUs) at ≥ 97% similarity with chimera filtering using the USEARCH algorithm [22]. The Ribosomal Database Project (RDP) classifier [23] was used to annotate the representative OTU sequences, and taxonomic information was obtained for each OTU. The OTUs table-based data were further visualized using Calypso [24].

### Statistical analysis

Statistical analyses were performed using SPSS 24.0 (IBM Company Headquarters, Chicago, IL), considering statistical significance when *P* < 0.05 (2-sided tests). All sow’s and piglet’s performance data were normally distributed and are reported as least square mean ± SEM. Gut microbiota data were non-normally distributed and were square root transformed, later presented as median. Microbiota statistical analysis was done with Calypso [24] for Shannon Index, Simpson’s index, ANOSIM, ANOVA and correlations. Normality of the data was analyzed with the Kolmogorov-Smirnov test and the Levene’s test was used to verify homogeneity of variance. In order to analyze which variables were associated with CY, we performed univariate analysis for all variables (including batches and parity). Interaction terms were tested and variables were included in the final model if *P* < 0.25. Backward stepwise elimination was used for final models. Sow data were subjected to linear regression with treatment (YD and CON) as fixed factor, and farrowing duration, total piglets born, live born piglets, level of progesterone at the beginning of farrowing and litter birth weight as covariates. Performance categories of sows and piglets used for Pearson’s correlation with fecal microbiota are presented in Table 1.

**Table 1:** Categorized variables used for the correlation analysis with Calypso.

Variables	Categories	Values
CY, g	high	≥3500
	low	< 3500
Colostrum IgG, mg/mL	high	> 51
	low	≤50
Colostrum IgM, mg/mL	high	≥5
	low	<5
Colostrum proteins, %	high	≥16
	low	<16
Progesterone (P_4_), ng/mL	high	≥4.51
	normal	<4.5
Farrowing duration (FD), min	long	>300
	normal	<300
Stillbirths	high	≥ 2
	normal	< 2
Average piglets BW_B_, g	normal	≥1400
	low	<1400
Piglets weight before weaning, g	bigger	>7500
	normal	5000–7500
	small	<5000
ADG, g	above average	<270
	average	180–270
	poor	<180

## Results

### Sow and litter performances

Reproductive performance values for the sows with and without YD are shown in Table 2.

**Table 2:** Effects of dietary yeast derivatives supplementation during gestation on sow reproductive performance (*P* values as an outcome of *t*-test).

Variable		CON (n = 19)	YD (n = 18)	SEM	*P*-value
Farrowing characteristics	Gestation length, days	116.4	116.0	0.2	0.39
	Sow parity	3.5	3.0	0.4	0.4
	Farrowing duration, min	**360.2**	**287.0**	**33.2**	**0.03**
	Litter size	13.8	15.6	0.8	0.14
	Live born piglets	**12.2**	**14.3**	**0.8**	**0.04**
	Stillborn piglets	1.6	1.3	0.3	0.65
	Birth interval, min	**37.1**	**22.4**	**4.5**	**0.03**
	Blood progesterone, ng/ml	**2.8**	**2.1**	**0.2**	**0.04**
Litter characteristics	Litter BW_B_ live born piglets, kg	17.8	19.5	1.2	0.31
	Average piglet BW_B_ live born piglets, kg	**1.4**	**1.3**	**0.01**	**0.001**
	Average piglet weight before weaning (6 ear tagged piglets), kg	7.6	8.0	0.34	0.54
	ADG (6 ear tagged piglets), g	223.3	226.0	10.3	0.15
	Average age at weight before weaning, days	**27.6**	**30.1**	**0.1**	**0.01**
	Piglet mortality (live born) at 24 h, %	5.1	2.8	1.2	0.24
	Piglet mortality at 4 weeks (6 ear tagged piglets), %	14.6	8.8	2.9	0.17
	CY, g	**3706**	**4581**	**296**	**0.04**
Colostrum characteristics	CY/live born piglets, g	**207**	**233**	**13.0**	**0.01**
	Fat %	**4.2**	**5.1**	**0.2**	**0.01**
	Protein %	16.5	17.3	0.5	0.29
	Lactose %	5.5	5.5	0.8	0.99
	Dry matter %	28.0	29.0	0.5	0.22
	IgG, mg/mL	62.3	65.2	3.1	0.51
	IgA, mg/mL	10.1	8.8	0.5	0.12
	IgM, mg/mL	4.9	4.3	0.3	0.25

### Colostrum yield, colostrum quality and composition

The YD supplementation of the sow diets had no effect on the IgG, IgA, IgM, and protein, lactose and dry matter percentage. However, sows fed the YD diets had significantly (*P <* 0.01) increased colostrum production. In addition, the fat percentage of colostrum of sows fed YD was increased (*P <* 0.01). Farrowing duration and level of progesterone were negatively associated with CY. A sow having a farrowing duration of 350 min or longer produced 451 g less colostrum compared with a sow with shorter farrowing duration (less than 350 min). Similarly, sows with a blood progesterone level of 4.5 ng/ml or higher produced 1571 g less colostrum compared with sows with normal (<4.5 ng/ml) blood progesterone levels during the start of farrowing (*P*<0.05). On the other hand, numbers of piglets born alive (per piglet) and live born litter birth weight (per gram) were associated with an increase of CY of 199.2 and 4.5 g respectively.

### DNA sequence data and bacterial community structure

After quality filtering as described above, a total of 101326, 156671, and 98688 DNA sequence reads were generated from sows, piglets at one week and piglet weaning samples, and reads were analyzed for assignment of OTUs (≥97% identity level). The diversity of the microbial communities in the different feeds and age groups was measured using Shannon and Simpson’s indices. The diversity indices showed the number of different taxa present in each sample; a higher number indicating greater diversity. The Shannon and Simpson indices for sows and piglets in the respective treatments are shown in S1 Fig.

**Fig 1:**
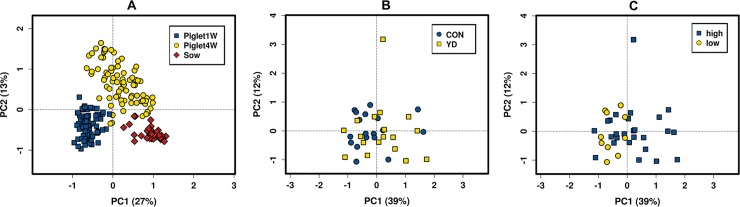
Bray-Curtis OUT’s level principal component analysis (PCA). (A) Individual fecal samples of sow, piglets at one week and four weeks of age. (B) Individual sow fecal samples in the control group and YD group. (C) Individual sow fecal samples based on the sow colostrum yield, high (≥ 3500 g) and low (< 3500 g).

Analysis of Similarity (ANOSIM), which compares the Bray-Curtis similarity between and within groups, was used to determine whether microbial population *beta* diversity differed between dietary treatment of sows, piglets at one week of age and piglets at four weeks of age. Irrespective of dietary treatment, sow, piglets at one week and piglets at four weeks of age were significantly different (*P* < 0.01), with a relatively higher corresponding *R*-value (0.677), suggesting that the sow and piglets at different ages were well separated from each other (S2 Fig). In contrast, although the ANOSIM of the Bray-Curtis similarity indicated that while the control fed sows and corresponding piglets, and YD fed sows and piglets differed (*P* = 0.03; *P* = 0.05; *P* = 0.009, sow, piglets at one week and piglets at four weeks respectively), the relative *R*-values suggest that the diet groups were not much separated from each other. However, the PCA analysis showed that microbiota of sows, piglets at one week and at four weeks of age differed considerably (Fig 1). In addition, PCA analysis also revealed the structure administration of gut microbiota. The YD administration differences were mainly for the first principal component (PC1), which accounted for the largest proportion (39%) of total variation (Fig 1). After the YD feeding during gestation, PCA indicated that different diets promoted the development of different gut microbial communities and therefore variation in the microbial communities in different CY groups (Fig 1)

**Fig 2:**
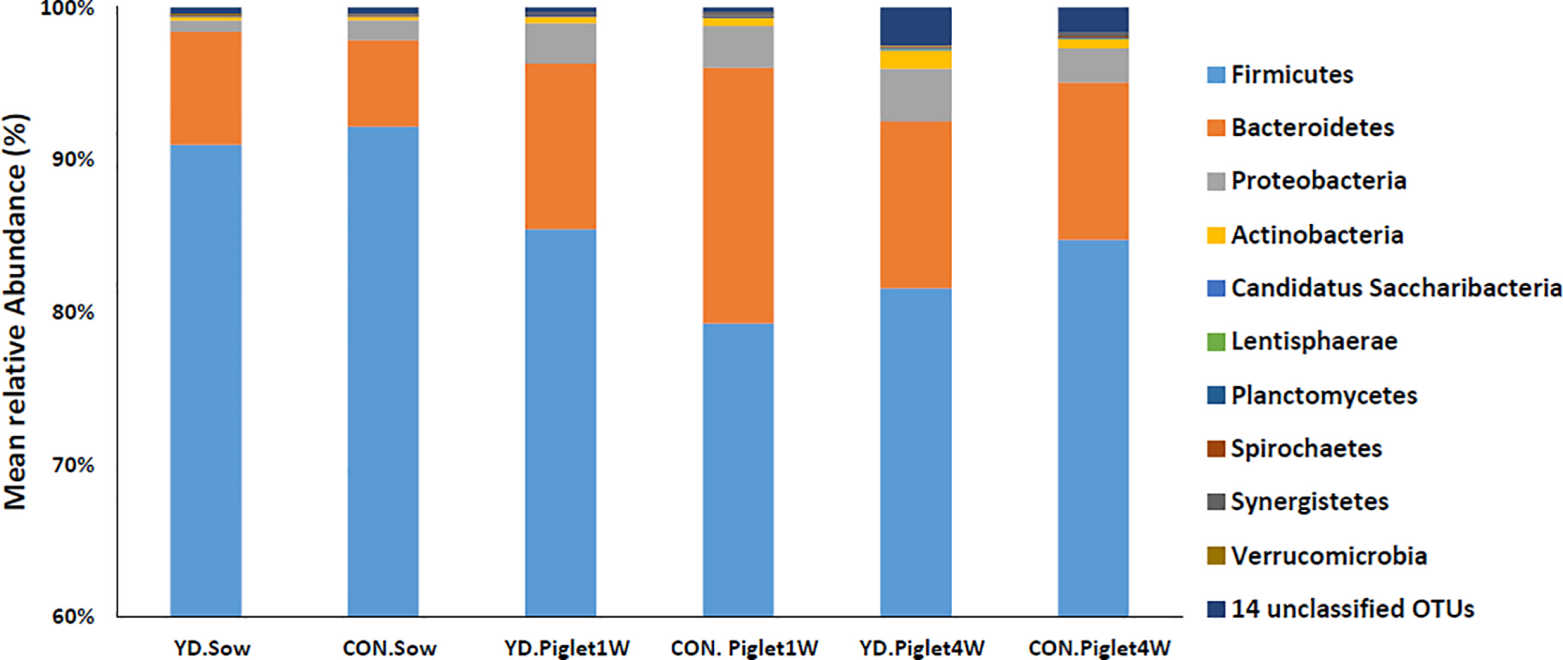
The distribution of bacterial phyla in fecal samples of sows and piglets of different ages fed different diets.

The results of the phylum distribution are shown in Fig 2 and S1 Table with all the statistical differences. Taxonomic assignment of the OTU identified 24 phyla in the fecal samples of the sows and piglets of different ages tested in this study. Two phyla, *Firmicutes* and *Bacteroidetes*, were dominant in the fecal samples regardless of diet treatment group (YD and CON) or time period of piglets samples. In the present study, after correction via a FDR calculation according to the Wilcoxon rank test, YD treatment profoundly decreased the *Proteobacteria* in sows. Piglets at one week of age raised with the sows fed with YD exhibited significantly increased abundance of *Firmicutes* and decreased abundance of *Bacteroidetes* compared with the control (S1 Table). However, piglets at 4 weeks of age raised with the sows fed with YD had significantly decreased abundances of *Spriochaetes* and *Synergistetes* and higher abundances of *Actinobacteria* and *Lentisphaerae* (S1 Table).

**Fig 3:**
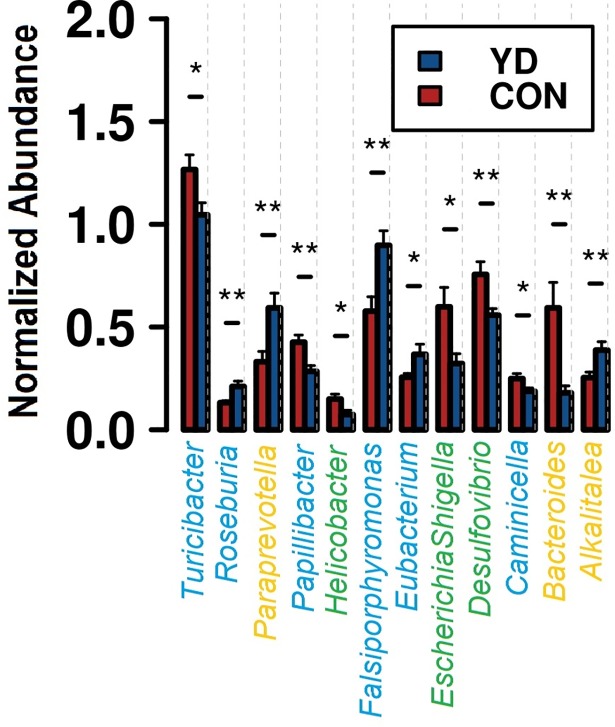
Microbiota profiles showing normalized square root transformed abundances of genera in two groups of sow. Genera are colored according to their phyla. *Firmicutes* (blue), *Bacteroides* (orange), and *Proteobacteria* (green). *P<0.05, **P<0.01.

**Fig 4:**
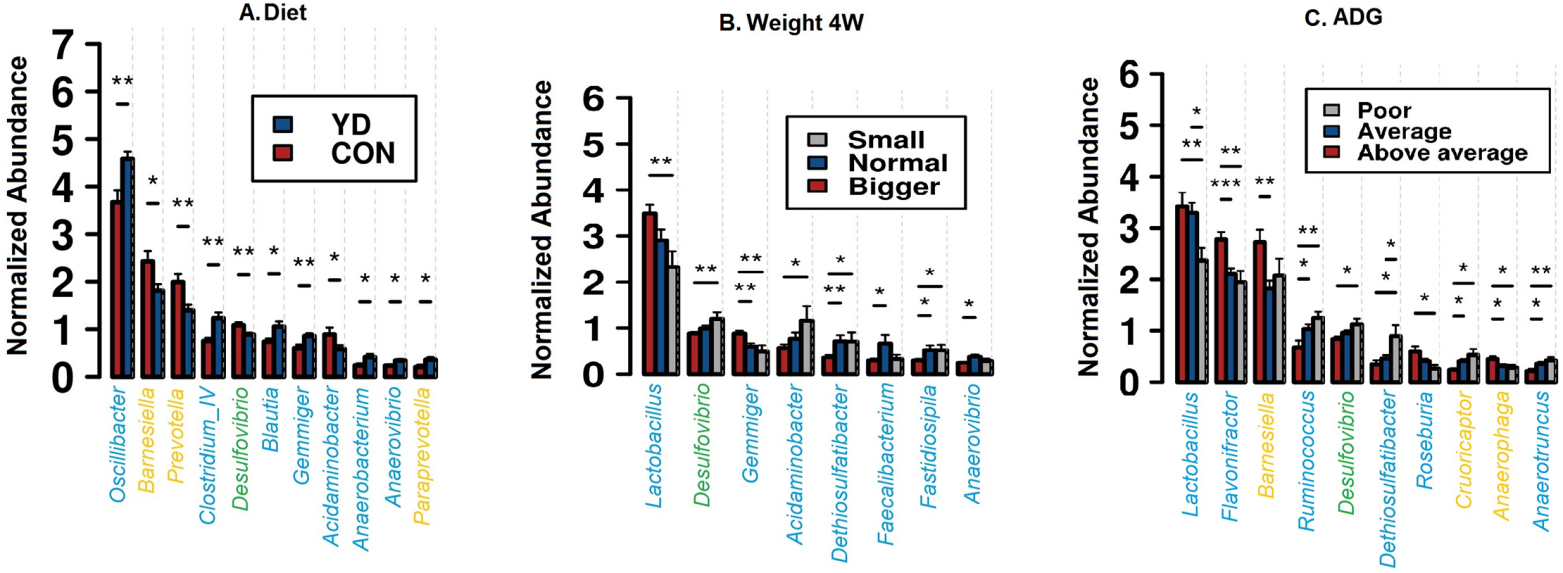
Microbiota profiles showing normalized square root transformed abundances of the genera in piglets at one week of age. (A) Diet: piglets raised with sow fed YD diet and CON diet. (B) Weight 4W: genera abundances at one week based on piglet weight at four weeks, large (>7500 g) normal (5000–7500 g) small (<5000 g). (C) ADG: genera abundances at one week based on ADG, above average (<270 g) average (180–270 g) poor (<180 g). Genera are colored according to their phyla. *Firmicutes* (blue), *Bacteroides* (orange), and *Proteobacteria* (green). *P<0.05, **P<0.01.

A total of 138 genera were identified from all the samples, regardless of diet, and sow and piglet age. *Romboutsia* was the dominant genus in sows, followed by *Clostridium sensu stricto*, *Lactobacillus*, *Oscillibacter*, *Intestinimonas*, *Sporobacter*, *Christensenella*, *Barnesiella*, *Flavonifractor*, *Terrisporobacter*, *Acidaminobacter*, *Lachnospiracea_incertae_sedis*, *and Turicibacter*, other genera being much less abundant (<1%) (S2 Table). A total of 12 genera differed between the dietary treatments of sows at genus level, including both abundant and less abundant genera (Fig 3). Feeding sows with YD resulted in increased abundance of *Roseburia*, *Paraprevotella*, *Falsiporphyromonas*, *Eubacterium* and *Alkalitalea* compared with the control. On the other hand, feeding sows with YD significantly decreased *Turicibacter*, *Papillibacter*, *Helicobacter*, *Escherichia/Shigella* and *Desulfovibrio*. In piglets at one week of age *Oscillibacter* was the dominant genus followed by *Lactobacillus*, *Sporobacter*, *Flavonifractor*, *Clostridium sensu stricto*, *Acetanaerobacterium*, *Barnesiella*, *Anaerovorax*, *Prevotella*, *Christensenella*, *Pseudoflavonifractor and Romboutsia* (<1%) (S3 Table). In addition, a total of 11 genera were identified among the 50 most abundant as differing between the two treatments groups at genus level, including three abundant (> 1%) and seven less abundant genera (Fig 4, all differentially abundant genera are listed in S4 Table). In piglets at one week of age, several changes were evident in the relative abundance of genera as a result of being raised by a sow fed with YD. Among the most abundant genera the proportion of *Barnesiella*, *Prevoteella*, *Desulfovibrio* and *Acidominobacter* were all significantly reduced at one week of age (*P* <0.01, Fig 4). One-week old piglets raised with the YD sows also had a significantly greater proportion of the genera *Oscillibacter*, *Clostridium IV*, *Blautia*, *Gemmiger*, *Anaerobacterium*, *Anaerovibrio* and *Paraprevotella* (Fig 4). However, *Romboutsia* was the most abundant genus in piglets at four weeks of age, followed by *Clostridium IV*, *Clostridium sensu stricto*, *Sporobacter*, *Oscillibacter*, *Barnesiella*, *Flavonifractor*, *Lactobacillus*, *Anaerovorax*, *Prevotella*, *Christensenella*, and *Acetanaerobacterium* (S5 Table).

### Correlation of gut microbiota with sow and litter performances

**Fig 5:**
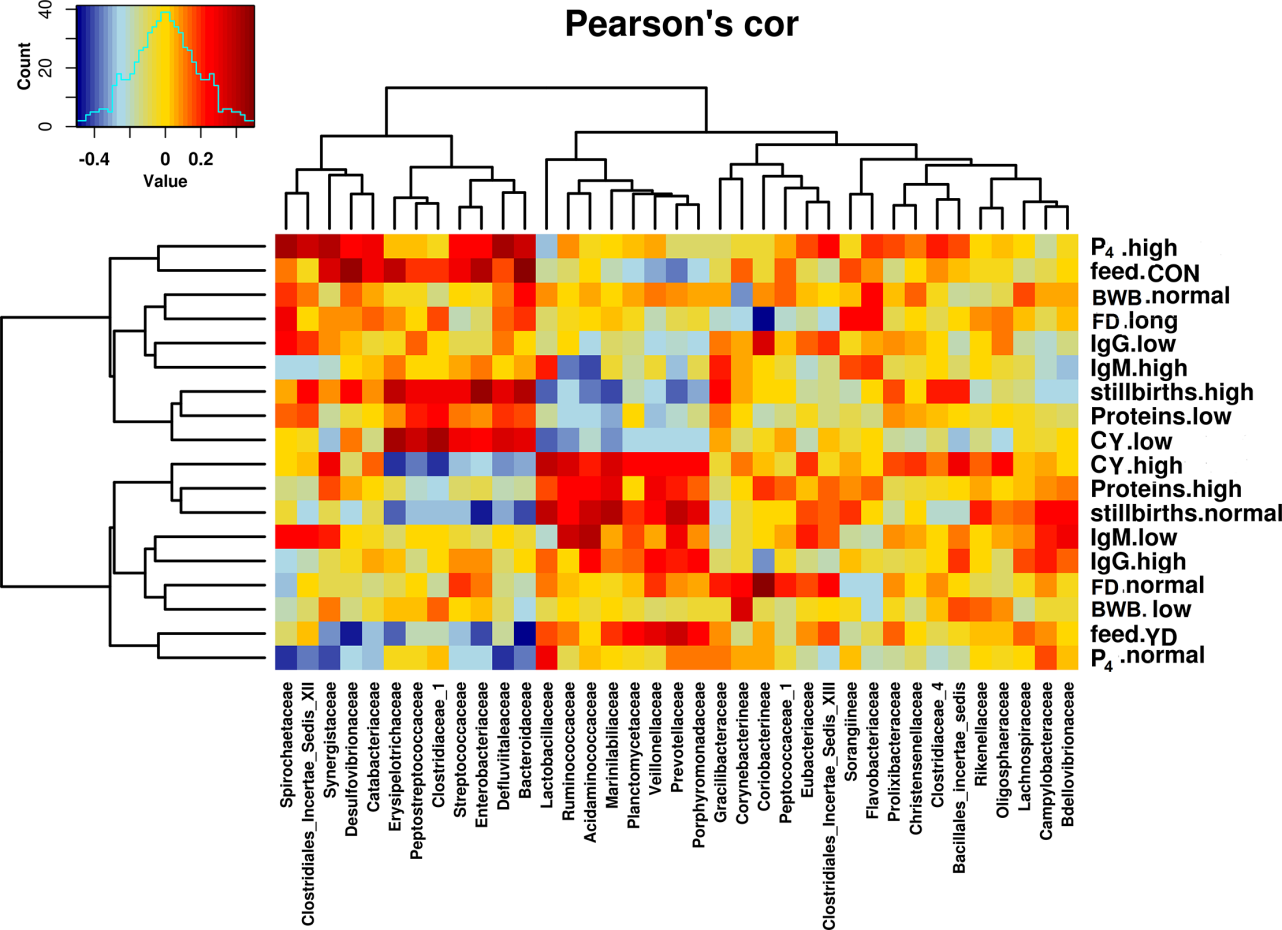
Heatmap showing correlation (based on Pearson’s test) between the normalized square root transformed abundance of microbiota family and the performance parameters of sows. Positive or negative correlations are represented by shades of *red or blue* respectively. Dendrograms represent different clustering among the different performance parameters and the microbiota families.

Some of the changes in the microbial abundances were correlated with the colostrum yield, colostrum IgG, IgM, blood progesterone level, farrowing duration and stillbirths as determined by the Pearson’s correlation heatmap analysis (Fig 5). High colostrum yield, high colostrum proteins, high colostrum IgG, normal blood progesterone level and normal farrowing duration clustered with YD feeding and were positively correlated to the bacterial families *Lacotobacillaceae*, *Ruminococcaceae*, *Acidaminococcaceae*, *Planctomycetaceae*, *Marinilabiliaceae*, *Veillonellaceae* and *Prevotellaceae* (Fig 5). On the other hand, feeding sows with CON, low colostrum yield, low colostrum proteins, low colostrum IgG, high level of blood progesterone and long farrowing duration clustered and were positively correlated with bacterial families *Erysipelotrichaceae*, *Peptostreptococcaceae*, *Colostridiaceae*, *Streptococcaceae*, *Enterobacteriaceae*, *Desulfovibrionaceae*, *Bacteroidaceae*, *Spirochaetaceae*, *Defluviitaleaceae*, *Synergistaceae* (Fig 5). In one week old piglets raised with YD fed sows, particular bacterial species were significantly more abundant than in piglets raised with control fed sows (Fig 4). Piglets growing faster and larger in size at four weeks of age had higher relative abundances of *Lactobacillus*, *Flavonifractor*, *Barnesiella*, *Gemmiger*, *Faecalibacterium*, *Roseburia and Anaerophaga* (Fig 4) at one week of age. On the other hand, piglets growing more slowly and with poor ADG had more *Desulfovibrio*, *Acidaminobacter*, *Dethiosulfatibacter*, *Fastiduisipila*, *Ruminnococcus* and *Anaerotruncus* (Fig 4) at one week of age (*P* <0.01).

## Discussion

To our knowledge, this is the first report presenting the results of high-throughput analysis of the effects of YD on the gut microbiota and its association with the production of sows and piglets. We used a 16SrRNA gene-based high-throughput sequencing approach to demonstrate a role of YD inclusion in the gestating diet in modulating the composition of the gut microbiota of sows and piglets. Previously published studies regarding YD effects on sow production and performance have been inconsistent. However, YD effects on sows are associated with growth stage [25] and YD structure and concentration [1,26]. Therefore, our study explored a specific acid hydrolyzed YD sow diet supplement to investigate the treatment effect on sow performance and subsequent litter performance and production.

In the present study, feeding sows a YD diet affected farrowing duration, average number of piglets born in total, live born piglets, birth interval and blood progesterone level around the start of farrowing. The duration of the whole farrowing process was affected by the YD diet and was clearly shorter than for the sows fed a control diet. Similarly, average birth interval of individual piglets was reduced in YD fed sows. Thus, the farrowing process was shorter because of faster birth of individual piglets. There is no previous literature available regarding the farrowing duration and YD supplementation in sows, present results might be explained by the higher drop in blood progesterone concentration in the YD fed sows. This better physiology of the sow, might be due to more nutrients available, and promotion of beneficial gut microbiota [27,28]. Among the control fed sows, 29% had higher progesterone than is considered “normal” at the beginning of farrowing. However, feeding high fiber diets reduced progesterone in cows [29], but no such effect was found in pigs [30]. Nonetheless, feeding sows with YD feed increased the number of total piglets born and live born piglets. A similar finding was reported by Czech *et al*. [31]. However, a few recent studies revealed that sow dietary YD supplementation during gestation had no effect on the average numbers of piglets born and those born alive [1,5,32]. It has been reported that live born litter size is mainly determined by the fertilization rate and embryonic mortality during early pregnancy [33], and therefore supplementing the sow diet with YD during gestation might not have had an effect on the litter size as we report. In addition, the average litter weights of sows fed YD were higher (*P* < 0.01), but there were no differences between the individual piglets. This results from the larger litter size of the sows fed YD. The growth performance and survival of the piglets depends on their BW_B_ and colostrum uptake [34,35] and early-life establishment of gut microbiota [36].

Sows fed with YD produced 23% more colostrum (*P* < 0.05) and resulted in 13% more colostrum consumption for the piglets (*P* < 0.01). Le Dividich *et al*. [37] reported that feeding sows with YD produced 15% more colostrum and therefore the colostrum intake of piglets was 12% higher. Colostrum is rich in various immunoglobulins and bioactive compounds, including growth-promoting factors, newborn piglets are without sufficient immune protection, and therefore they are highly dependent on maternal colostrum-derived immunoglobulins [38]. Supplementing the sow diet with YD did not affect IgG, IgA and IgM contents of colostrum, as previously reported [37]. However, Duan *et al*. [1] reported a higher concentration of IgM in YD fed sows and O’Quinn *et al*. [4] reported increased concentrations of IgG, IgA, and IgM in prenursing colostrum of YD-treated sows. The colostrum fat was increased by 21% (*P*<0.01), but there were no significant increases in other milk components (protein, lactose and DM). The amount and composition of colostrum produced by the sow can be influenced by sow and litter characteristics, endocrine status, nutrition, environmental factors or a combination of these factors [7,17]. Therefore, YD might have also been associated with beneficial gut microbiota development (*Roseburia*, *Ebacterium*, *Paraprevotella*) positive immune stimulation, which may have improve the colostrum components.

Many studies have demonstrated that certain microorganisms can exert beneficial effects on the sow and thus boost the production performance [25,36,39]. Therefore, we investigated the effect of YD on fecal microbiota of sows, colostrum yield, colostrum composition, physiology and piglet growth. The YD can influence the intestinal microbiota and therefore also the sow’s immune status by activation of alternative complement pathway, release of lysosomal enzymes, binding to the specific receptor of macrophages and cytokine [12–15]. The PCA analysis showed that sow microbiota was significantly influenced by the administration of YD, changing the gut microbiota composition at the phylum level. In the present study, a lower relative abundance of *Proteobacteria* was observed in the YD group, which can be considered beneficial because increased prevalence of *Proteobacteria* is a marker for an unstable microbial community (dysbiosis), a potential diagnostic criterion for diseases [40] and is also linked with intestinal inflammation [41]. This phylum includes bacteria known to cause intestinal pathology in humans and animals [42,43]. Interestingly, the increased abundance of *Proteobacteria* at family levels (*Enterobacteriaceae*, *Desulfovibrionaceae*, *Desulfovibrionaceae*) in the CON diet group was associated with negative performance parameters such as low colostrum yield, low colostrum proteins, low colostrum IgM, and high stillbirth numbers.

Studies show that in the presence of mannan-oligosaccharides from YD the enteric pathogen attaches to the mannan compounds in the gut lumen instead of the gut epithelia, which reduces colonization of the gut [8–11]. However, the efficiency of the mannan products is highly subject to their chemical structure [25]. We established that YD addition reduced several genera that were previously reported to cause clinical and subclinical infection in humans and animals. *Turicibacter*, *Desulfovibrio*, *Papillibacter*, *Helicobacter* and *Escherichia/Shigella* were the most abundant in control fed sows. *Turicibacter* is a putative pathogen, and it is possible that when present in the pig may cause subclinical infections or have other deleterious effects on the GI tract [44]. *Desulfovibrio* bacteria are associated with inflammatory intestine syndrome in humans and animals, and their metabolic end product, hydrogen sulfide, is a cytotoxic compound [45–48]. This compound may act through an inhibition of butyrate oxidation, the main energy source for colonocytes. The impairment of the functions of the intestinal epithelium would lead to cell death and chronic inflammation [45]. The gut microbiota exists in a dynamic state, increasing the number of any bacterial species may result in the decrease of another species. Some studies show that the reduction in the number of pathogenic bacteria in response to dietary YD supplementation is associated with an increase in beneficial microbiota [9,49,50]. Feeding YD to sows, which in turn may modify the substrate availability and physiological conditions of the gastrointestinal tract [e.g. fermentation products, luminal pH and bile acid concentration [51]] were probably the main cause of increased abundances of *Paraprevotella*, *Roseburia*, *Ebacterium* and *Alkalitalea*. *Paraprevotella*, *Roseburia* are the intestinal microbes that are able to degrade cellulose and hemicellulose [52,53]. However, *Roseburia* and *Ebacterium* produce butyric acid, the main energy source for the colonocytes and protects from inflammation [54–56]. Thus, it can be speculated that, in response to YD, the gut microbiota may contribute to the host metabolism, hydrolyze the feed and promote nutrition absorption, which could have led to the increased CY and colostrum functional components and resulted in the positive correlation.

PCA analysis revealed that piglets at one week of age have unique microbiota. Many studies, however, report similar results, that the suckling piglet has unique microbiota acquired from the mother. The maternal dietary treatment impacted the composition of the microbiota in piglets, which was distinct from the sow’s fecal microbial alterations. This was also observed when feeding sows with inulin, prebiotics or probiotics [57,58]. Moreover, the piglet acquires not only the fecal microbiota from the sow but also microbial communities present in the birth canal, on the skin and from the environment around the mother. Furthermore, besides a fecal transfer, the colostrum and the chemical and microbial composition of milk might also influence the intestinal microbiota of the progeny. This merits further investigation. Nuria *et al*. [36] reported that *Desulfovibrio* was shared between mother and piglets. *Lactobacillus*, *Eubacterium* and *Clostridium* are among other bacteria identified that could be transferred from mother to piglet gut via feces or suckling. In humans, *Lactobacillus fermentum* has been confirmed to represent vertical transfer between mother and neonate via breast milk [59]. Irrespective of the treatment, piglets growing faster and being heavier at four weeks of age had higher abundances of *Lactobacillus* at one week of age. Interestingly, different species of *Lactobacillus* have been used as a growth-promoting feed supplement preventing and treating diarrhea in piglets and maximizing the average daily gain, crude protein apparent digestibility and serum specific IgG level [60]. Thus, it is likely that *Lactobacillus* from the mother’s gastrointestinal tract or milk may colonize the piglet’s gastrointestinal tract and promote piglet growth. However, *Flavonifractor*, *Barnesiella* and *Roseburia* have a positive influence on the daily growth of the piglets [36]. Piglets belonging to the YD fed sows had higher colostrum consumption, which could be a reason for observed relative abundance of *Oscillibacter*, *Clostridium IV*, *Blautia* and *Gemmiger* in their gastrointestinal tracts. It has been suggested that species from these genera are abundant in the neonate gastrointestinal microbiota because they are adapted to utilize a wide range of milk oligosaccharides as a unique carbon source [61–63].

Overall, this study demonstrated that addition of YD in the gestation diet was able to enhance colostrum production, and especially colostrum fat, ensuring enhanced colostrum intake per kg live born piglets, more energy and sustained piglet immunity. Supplementing the gestation diet with YD may change the gut microbiota, alleviate farrowing stress, improve sow physiology and, therefore, produce more viable piglets with a stable gut.

## Supporting information

S1 Fig Strip chart showing diversity measures (Simpson’s and Shannon’s indices) between groups at the OUT level. (A) Shannon Index for sows fed YD and control group. (B) Shannon Index for piglets at one week of age with two different sow treatment groups. (C) Shannon Index for piglets at four weeks of age with two different sow treatment groups. (D) Simpson’s Index for sows fed YD and control group. (E) Simpson’s Index for piglets at one week of age with two different sow treatment groups. (F) Simpson’s Index for piglets at four weeks of age with two different sow treatment groups.
(TIF)

S2 Fig Notched boxplot representation of ANOSIM analysis with dissimilarity based on Bray-Curtis distance. Sample dissimilarity within each group is indicated in the graph and overall dissimilarity is indicated as “Between”. (A) Sow, piglets at one week and piglets at four weeks of age. (B) Sow treatment groups. (C) Piglets at one week of age with two different sow treatment groups. (D) Piglets at four weeks of age with two different sow treatment groups.
(TIF)

S1 Text Ingredient and nutrient composition of sow basal diet and piglet creep feed. (DOCX)

S2 Text Biochemical properties and chemical composition of tested YD (Progut®). (DOCX)

S3 Text Partial Illumina TruSeq adapter sequences added to the 5’ ends. (DOCX)

S1 Table Abundant phyla in different sows and piglet treatment groups. *P* values are based on the results from the Mann-Whitney test.
(DOCX)

S2 Table 20 most abundant genera in sows. Values are presented in normalized square root transformed abundance. *P* values are based on the results from the Mann-Whitney test.
(DOCX)

S3 Table 20 most abundant genera in piglets at one week of age. Values are presented in normalized square root transformed abundance. *P* values are based on the results from the Mann-Whitney test.
(DOCX)

S4 Table Differentially abundant genera in piglets at one week of age. Values are presented in normalized square root transformed abundance. *P* values are based on the results from the Mann-Whitney test.
(DOCX)

S5 Table 20 most abundant genera in piglets at four weeks of age. Values are presented in normalized square root transformed abundance. P values are based on the results from the Mann-Whitney test.
(DOCX)
