# Supplementary figures and images for: Dietary supplementation with yeast hydrolysate in pregnancy influences colostrum yield and gut microbiota of sows and piglets after birth
Source: PLoS One. 2018 May 24;13(5):e0197586. doi: 10.1371/journal.pone.0197586 (PMC5967808; doi:10.1371/journal.pone.0197586)

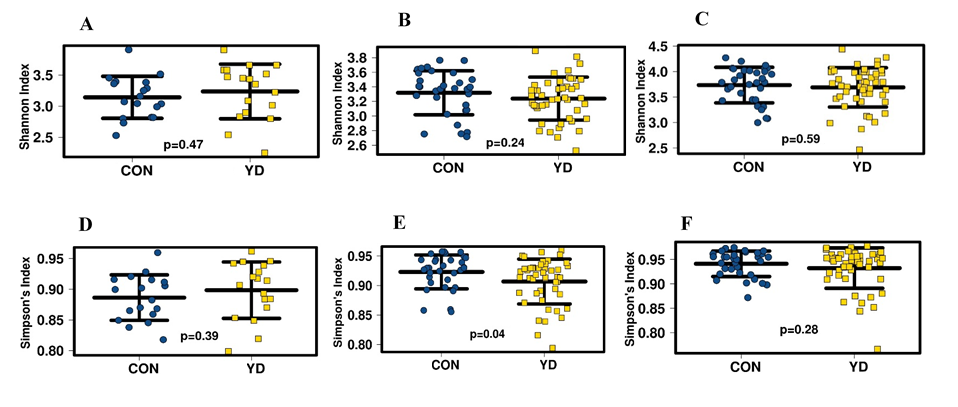

Supplement: S1 Fig — (A) Shannon Index for sows fed YD and control group. (B) Shannon Index for piglets at one week of age with two different sow treatment groups. (C) Shannon Index for piglets at four weeks of age with two different sow treatment groups. (D) Simpson’s Index for sows fed YD and control group. (E) Simpson’s Index for piglets at one week of age with two different sow treatment groups. (F) Simpson’s Index for piglets at four weeks of age with two different sow treatment groups. (TIF) [file pone.0197586.s001.tif]

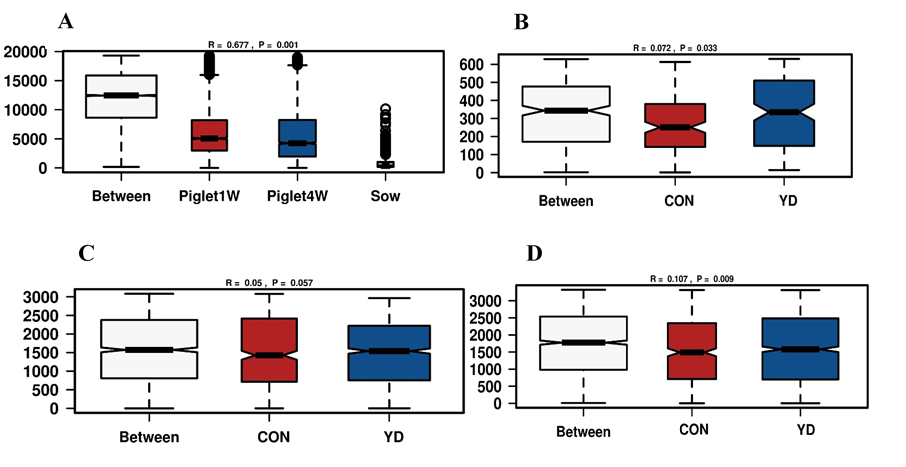

Supplement: S2 Fig — Sample dissimilarity within each group is indicated in the graph and overall dissimilarity is indicated as “Between”. (A) Sow, piglets at one week and piglets at four weeks of age. (B) Sow treatment groups. (C) Piglets at one week of age with two different sow treatment groups. (D) Piglets at four weeks of age with two different sow treatment groups. (TIF) [file pone.0197586.s002.tif]
