# Supplementary material for: Dietary supplementation with yeast hydrolysate in pregnancy influences colostrum yield and gut microbiota of sows and piglets after birth
Source: PLoS One. 2018 May 24;13(5):e0197586. doi: 10.1371/journal.pone.0197586 (PMC5967808; doi:10.1371/journal.pone.0197586)
Supplement: S1 Text — (DOCX) [file pone.0197586.s003.docx]

| Raw material (%) | Lactation diet | Gestation diet |
| --- | --- | --- |
| Dehulled oats | 25.81 | 2.0 |
| Wheat | 20 | 5.0 |
| Oats | 13.4 | 19.31 |
| Wheat feed meal | 8.0 | 7.0 |
| Barley | 8.0 | 21.5 |
| Soyabean meal | 8.0 | 4.2 |
| Sugarbeet pulp | 3.0 | 5.0 |
| Sunflower meal | 3.0 | 2.0 |
| Wheat bran | 3.0 | 15.0 |
| Vegetable oils and fatty acids | 2.7 | 0.5 |
| Premixes | 1.83 | 1.76 |
| Calcium carbonate | 1.31 | 1.51 |
| Amino acids | 0.8 | 0.31 |
| Monocalcium phosphate | 0.66 | 0.3 |
| Salt | 0.49 | 0.25 |
| Oat hull meal | - | 9.0 |
| Molasses | - | 2.0 |
| Pea | - | 2.0 |
| Feed fiber | - | 1.0 |
| Sodiumbicarbonate | - | 0.36 |
|  | | |
| Analysis (per kg) |  |  |
| Moisture (%) | 12.5 | 12.5 |
| Crude protein (%) | 15.8 | 12.8 |
| Crude fat (%) | 6.2 | 3.5 |
| Crude fiber (%) | 5.6 | 10.0 |
| Ash (%) | 6.1 | 6.2 |
| Methionine (g) | 3.1 | 2.1 |
| Lysine (g) | 9.6 | 6.5 |
| Calcium (g) | 9.5 | 7.5 |
| Phosphorus (g) | 6.1 | 5.3 |
| Sodium (g) | 2.0 | 2.3 |
| NE (MJ) | 10.1 | 8.0 |

Table A. Sow diet composition

Table B. composition of piglet's creep feed

| Raw material (%) | Piglet's creep feed |
| --- | --- |
| Dehulled oats | 27.93 |
| Wheat | 24.0 |
| Whey meal | 20.5 |
| Barley | 12.3 |
| Potato protein | 2.3 |
| Soya protein concentrate | 1.0 |
| Premixes | 3.04 |
| Glucose | 2.0 |
| Linseed expeller | 1.5 |
| Amino acids | 1.4 |
| Fish meal | 1.5 |
| Vegetable oils and fatty acids | 0.5 |
| Calcium carbonate | 0.4 |
| Monocalcium phosphate | 1.4 |
| Salt | 0.23 |
|  | |
| Analysis (per kg) |  |
| Moisture (%) | 12.0 |
| Crude protein (%) | 19.0 |
| Crude fat (%) | 7.9 |
| Crude fiber (%) | 2.6 |
| Ash (%) | 6.5 |
| Methionine (g) | 5.4 |
| Lysine (g) | 14.8 |
| Calcium (g) | 9.0 |
| Phosphorus (g) | 7.0 |
| Sodium (g) | 2.2 |
| NE (MJ) | 11.0 |
