# Supplementary material for: Dietary supplementation with yeast hydrolysate in pregnancy influences colostrum yield and gut microbiota of sows and piglets after birth
Source: PLoS One. 2018 May 24;13(5):e0197586. doi: 10.1371/journal.pone.0197586 (PMC5967808; doi:10.1371/journal.pone.0197586)
Supplement: S2 Text — (DOCX) [file pone.0197586.s004.docx]

**Biochemical properties**

Progut®, a processed yeast hydrolysate (*Saccharomyces cerevisiae*). It’s patented (EP 1387620) and produced by strong acid hydrolysis process in pH 2.4 -2.6 for 4 hours. The soluble extract fraction is not removed, and the product contains all bioactive elements of a yeast cell. This results in higher water solubility (> 50 %) than in standard brewery yeast or in yeast cell wall products, and higher content of soluble mannose and betaglucan structures. The total mannose and betaglucan contents of the product are 5-10%.

**Ingredients**

| Yeast hydrolysate  - *Saccharomyces cerevisiae* | 91% |
| --- | --- |
| Salt  - Sodiumphosphate | 5% |
| Anticaking agent  - Sepiolite (E562) | 4% |

**Chemical properties**

| Moisture | 6.0 % (≤7.0 %) |
| --- | --- |
| Crude protein | 39.0 % (36.0 – 45.0 %) |
| Ash | 18.5 % (16.2 – 20.8 %) |
| Crude fat | 2.0 % |
| Crude fiber | 0.2 % |
| Non-fibre extracts | 34.3 % |
| Calcium (Ca) | 0.2 % |
| Phosphorus (P) | 3.7 % |
| Sodium (Na) | 1.3 % |
| Amino acids (%) as fed: | |
| Lysine | 1.6 % |
| Methionine + cysteine | 1.3 % |
| Threonine | 1.2 % |
