# Supplementary material for: Dietary supplementation with yeast hydrolysate in pregnancy influences colostrum yield and gut microbiota of sows and piglets after birth
Source: PLoS One. 2018 May 24;13(5):e0197586. doi: 10.1371/journal.pone.0197586 (PMC5967808; doi:10.1371/journal.pone.0197586)
Supplement: S3 Text — (DOCX) [file pone.0197586.s005.docx]

| Forward | Reverse |
| --- | --- |
| F_1; ATCTACACTCTTTCCCTACACGACGCTCTTCCGATCT | R_1; GTGACTGGAGTTCAGACGTGTGCTCTTCCGATCTGACT |
| F_2; ATCTACACTCTTTCCCTACACGACGCTCTTCCGATCTgt | R_2; GTGACTGGAGTTCAGACGTGTGCTCTTCCGATCTa |
| F_3; ATCTACACTCTTTCCCTACACGACGCTCTTCCGATCTagag | R_3; GTGACTGGAGTTCAGACGTGTGCTCTTCCGATCTtct |
| F_4; ATCTACACTCTTTCCCTACACGACGCTCTTCCGATCTtagtgt | R_4; GTGACTGGAGTTCAGACGTGTGCTCTTCCGATCTctgagtg |
