# Supplementary material for: Dietary supplementation with yeast hydrolysate in pregnancy influences colostrum yield and gut microbiota of sows and piglets after birth
Source: PLoS One. 2018 May 24;13(5):e0197586. doi: 10.1371/journal.pone.0197586 (PMC5967808; doi:10.1371/journal.pone.0197586)
Supplement: S1 Table — P values are based on the results from the Mann-Whitney test. (DOCX) [file pone.0197586.s006.docx]

| Taxa (Phylum) | Sow | | | Piglets one Week | | | Piglets four Weeks | | |
| --- | --- | --- | --- | --- | --- | --- | --- | --- | --- |
|  | CON | YD | *P*-value | CON | YD | *P*-value | CON | YD | *P*-value |
| Actinobacteria | 0.23 ± 0.15 | 0.21 ± 0.06 | 0.06 | 0.44 ± 0.25 | 0.32 ± 0.28 | 0.098 | 0.51 ± 0.56 | 0.73 ± 0.83 | 0.006 |
| Bacteroidetes | 4.57 ± 2.055 | 6.20 ± 5.63 | 0.14 | 15.07± 12.97 | 11.13 ± 8.17 | 0.003 | 8.81±6.06 | 8.77 ± 8.94 | 0.71 |
| Firmicutes | 93.23 ± 3.24 | 92.40 ± 6.00 | 0.25 | 82.0 ± 15.54 | 86.13 ± 9.66 | 0.001 | 86.87 ± 8.62 | 84.63 ± 13.51 | 0.13 |
| Lentisphaerae | 0.012 ± 0.02 | 0.012 ± 0.02 | 0.31 |  |  |  | 0.001 ± 0.02 | 0.007 ± 0.07 | 0.02 |
| Planctomycetes | 0.026 ± 0.03 | 0.038 ± 0.07 | 0.03 | 0.019 ± 0.10 | 0.048 ± 0.10 | 0.37 | 0.038 ± 0.07 | 0.031 ± 0.09 | 0.12 |
| Proteobacteria | 1.37 ± 0.22 | 0.49 ± 0.09 | 0.002 | 1.80 ± 2.28 | 1.79 ± 1.51 | 0.92 | 1.41 ± 1.55 | 1.60 ± 2.34 | 0.17 |
| Spirochaetes | 0.014 ± 0.06 | 0.0098 ± 0.02 | 0.48 |  |  |  | 0.0.62 ± 0.11 | 0.016 ± 0.05 | 0.01 |
| Synergistetes | 0.031 ± 0.03 | 0.027 ± 0.03 | 0.31 | 0.083 ± 0.30 | 0.050 ± 0.05 | 0.20 | 0.11 ± 0.21 | 0.050 ± 0.070 | 0.001 |
| Verrucomicrobia | 0.038 ± 0.03 | 0.067 ± 0.12 | 0.11 |  |  |  | 0.023 ± 0.04 | 0.027 ± 0.06 | 0.15 |
| Candidatus Saccharibacteria |  |  |  |  |  |  | 0.002 ± 0.01 | 0.003 ± 0.01 | 0.20 |
