# Supplementary material for: Dietary supplementation with yeast hydrolysate in pregnancy influences colostrum yield and gut microbiota of sows and piglets after birth
Source: PLoS One. 2018 May 24;13(5):e0197586. doi: 10.1371/journal.pone.0197586 (PMC5967808; doi:10.1371/journal.pone.0197586)
Supplement: S2 Table — P values are based on the results from the Mann-Whitney test. (DOCX) [file pone.0197586.s007.docx]

| Taxa (genus) | *P* | FDR | CON mean | YD mean | CON median | YD median |
| --- | --- | --- | --- | --- | --- | --- |
| *Romboutsia* | 0.17 | 0.57 | 4.87 | 4.53 | 4.97 | 4.56 |
| *Clostridium sensu stricto* | 0.27 | 0.68 | 4.8 | 4.4 | 4.94 | 4.43 |
| *Lactobacillus* | 0.27 | 0.68 | 2.31 | 2.83 | 2.04 | 2.56 |
| *Oscillibacter* | 0.59 | 0.92 | 2.1 | 2.19 | 2.08 | 2.14 |
| *Intestinimonas* | 0.68 | 0.92 | 1.92 | 2.01 | 1.79 | 1.94 |
| *Sporobacter* | 0.87 | 0.97 | 2 | 1.99 | 1.99 | 2.04 |
| *Christensenella* | 0.97 | 0.97 | 1.76 | 1.77 | 1.79 | 1.8 |
| *Barnesiella* | 0.16 | 0.57 | 1.47 | 1.7 | 1.44 | 1.62 |
| *Flavonifractor* | 0.7 | 0.92 | 1.38 | 1.41 | 1.36 | 1.44 |
| *Terrisporobacter* | 0.95 | 0.97 | 1.38 | 1.32 | 1.31 | 1.31 |
| *Acidaminobacter* | 0.74 | 0.92 | 1.25 | 1.3 | 1.08 | 1.27 |
| *Lachnospiracea incertae sedis* | 0.51 | 0.92 | 1.16 | 1.26 | 1.09 | 1.26 |
| *Turicibacter* | 0.011 | 0.22 | 1.27 | 1.05 | 1.33 | 1.03 |
| *Prevotella* | 0.097 | 0.57 | 0.69 | 0.95 | 0.56 | 0.86 |
| *Gracilibacter* | 0.96 | 0.97 | 0.87 | 0.9 | 0.86 | 0.84 |
| *Anaerovorax* | 0.13 | 0.57 | 0.78 | 0.88 | 0.7 | 0.87 |
| *Clostridium IV* | 0.72 | 0.92 | 0.86 | 0.88 | 0.79 | 0.89 |
| *Acetanaerobacterium* | 0.38 | 0.84 | 0.91 | 0.79 | 0.9 | 0.8 |
| *Alkalibacter* | 0.47 | 0.92 | 0.82 | 0.77 | 0.86 | 0.76 |
| *Clostridium XlVa* | 0.16 | 0.57 | 0.75 | 0.73 | 0.66 | 0.74 |
