# Supplementary material for: Dietary supplementation with yeast hydrolysate in pregnancy influences colostrum yield and gut microbiota of sows and piglets after birth
Source: PLoS One. 2018 May 24;13(5):e0197586. doi: 10.1371/journal.pone.0197586 (PMC5967808; doi:10.1371/journal.pone.0197586)
Supplement: S3 Table — Values are presented in normalized square root transformed abundance. P values are based on the results from the Mann-Whitney test. (DOCX) [file pone.0197586.s008.docx]

| Taxa (genus) | *P* | FDR | CON mean | YD mean | CON median | YD median |
| --- | --- | --- | --- | --- | --- | --- |
| *Oscillibacter* | 0.0021 | 0.036 | 3.67 | 4.58 | 3.93 | 4.57 |
| *Lactobacillus* | 0.9 | 0.91 | 3.14 | 3.21 | 3.15 | 3.48 |
| *Sporobacter* | 0.83 | 0.91 | 2.6 | 2.63 | 2.67 | 2.59 |
| *Flavonifractor* | 0.48 | 0.8 | 2.24 | 2.2 | 2.38 | 2.16 |
| *Clostridium sensu stricto* | 0.39 | 0.78 | 2.42 | 2.15 | 2.3 | 2.06 |
| *Acetanaerobacterium* | 0.64 | 0.81 | 1.85 | 2.03 | 2.08 | 2.01 |
| *Barnesiella* | 0.025 | 0.083 | 2.43 | 1.81 | 2.31 | 1.74 |
| *Anaerovorax* | 0.91 | 0.91 | 1.49 | 1.45 | 1.43 | 1.39 |
| *Prevotella* | 0.011 | 0.055 | 1.99 | 1.4 | 1.88 | 1.23 |
| *Christensenella* | 0.56 | 0.8 | 1.37 | 1.36 | 1.42 | 1.22 |
| *Pseudoflavonifractor* | 0.097 | 0.22 | 1.15 | 1.35 | 1.05 | 1.29 |
| *Romboutsia* | 0.09 | 0.22 | 1.47 | 1.29 | 1.41 | 1.17 |
| *Clostridium XlVa* | 0.016 | 0.064 | 1.58 | 1.27 | 1.37 | 1.06 |
| *Clostridium IV* | 0.0036 | 0.036 | 0.75 | 1.23 | 0.62 | 1.01 |
| *Blautia* | 0.053 | 0.15 | 0.74 | 1.06 | 0.66 | 0.79 |
| *Escherichia/Shigella* | 0.5 | 0.8 | 0.93 | 1.04 | 0.7 | 0.96 |
| *Ruminococcus* | 0.56 | 0.8 | 0.97 | 1.03 | 0.75 | 0.85 |
| *Intestinimonas* | 0.8 | 0.91 | 1 | 0.99 | 0.96 | 0.95 |
| *Desulfovibrio* | 0.0093 | 0.055 | 1.08 | 0.89 | 1 | 0.89 |
| *Alistipes* | 0.65 | 0.81 | 0.77 | 0.8 | 0.76 | 0.84 |
