# Supplementary material for: Dietary supplementation with yeast hydrolysate in pregnancy influences colostrum yield and gut microbiota of sows and piglets after birth
Source: PLoS One. 2018 May 24;13(5):e0197586. doi: 10.1371/journal.pone.0197586 (PMC5967808; doi:10.1371/journal.pone.0197586)
Supplement: S4 Table — Values are presented in normalized square root transformed abundance. P values are based on the results from the Mann-Whitney test. (DOCX) [file pone.0197586.s009.docx]

| Taxa (genus) | *P* | FDR | CON mean | YD mean | CON median | YD median |
| --- | --- | --- | --- | --- | --- | --- |
| *Cloacibacillus* | 0.00027 | 0.026 | 0.31 | 0.13 | 0.2 | 0.05 |
| *Oscillibacter* | 0.0021 | 0.088 | 3.67 | 4.58 | 3.93 | 4.57 |
| *Gemmiger* | 0.0034 | 0.088 | 0.6 | 0.86 | 0.5 | 0.89 |
| *Clostridium IV* | 0.0036 | 0.088 | 0.75 | 1.23 | 0.62 | 1.01 |
| *Bacteroides* | 0.0048 | 0.094 | 0.7 | 0.55 | 0.58 | 0.42 |
| *Mogibacterium* | 0.0061 | 0.1 | 0.11 | 0.2 | 0.075 | 0.17 |
| *Desulfovibrio* | 0.0093 | 0.12 | 1.08 | 0.89 | 1 | 0.89 |
| *Gracilibacter* | 0.01 | 0.12 | 0.19 | 0.33 | 0.16 | 0.25 |
| *Prevotella* | 0.011 | 0.12 | 1.99 | 1.4 | 1.88 | 1.23 |
| *Sutterella* | 0.013 | 0.12 | 0.18 | 0.1 | 0.11 | 0.07 |
| *Bilophila* | 0.013 | 0.12 | 0.24 | 0.17 | 0.24 | 0.14 |
| *Dietzia* | 0.015 | 0.12 | 0.044 | 0.079 | 0.015 | 0.07 |
| *Clostridium XlVa* | 0.016 | 0.12 | 1.58 | 1.27 | 1.37 | 1.06 |
| *Terrisporobacter* | 0.023 | 0.14 | 0.26 | 0.15 | 0.17 | 0.13 |
| *Cruoricaptor* | 0.023 | 0.14 | 0.34 | 0.43 | 0.24 | 0.37 |
| *Caminicella* | 0.023 | 0.14 | 0.19 | 0.28 | 0.15 | 0.27 |
| *Anaerofilum* | 0.024 | 0.14 | 0.18 | 0.26 | 0.14 | 0.21 |
| *Barnesiella* | 0.025 | 0.14 | 2.43 | 1.81 | 2.31 | 1.74 |
| *Macellibacteroides* | 0.028 | 0.14 | 0.09 | 0.15 | 0 | 0.09 |
| *Subdoligranulum* | 0.032 | 0.16 | 0.47 | 0.78 | 0.16 | 0.4 |
| *Anaerobacterium* | 0.035 | 0.16 | 0.24 | 0.41 | 0.16 | 0.32 |
| *Catabacter* | 0.041 | 0.18 | 0.15 | 0.22 | 0.11 | 0.19 |
| *Lactivibrio* | 0.043 | 0.18 | 0.19 | 0.23 | 0.18 | 0.21 |
| *Defluviitalea* | 0.044 | 0.18 | 0.32 | 0.24 | 0.3 | 0.23 |
| *Paraprevotella* | 0.049 | 0.19 | 0.2 | 0.36 | 0.09 | 0.22 |
| *Blautia* | 0.053 | 0.2 | 0.74 | 1.06 | 0.66 | 0.79 |
| *Allisonella* | 0.071 | 0.26 | 0.12 | 0.016 | 0 | 0 |
| *Bulleidia* | 0.074 | 0.26 | 0.014 | 0.047 | 0 | 0 |
| *Dorea* | 0.08 | 0.26 | 0.42 | 0.47 | 0.4 | 0.48 |
| *Anaerovibrio* | 0.086 | 0.26 | 0.23 | 0.34 | 0.23 | 0.28 |
| *Parabacteroides* | 0.087 | 0.26 | 0.62 | 0.5 | 0.58 | 0.43 |
| *Lachnospiracea incertae sedis* | 0.087 | 0.26 | 0.21 | 0.15 | 0.15 | 0.12 |
| *Phascolarctobacterium* | 0.089 | 0.26 | 0.71 | 0.79 | 0.7 | 0.8 |
| *Romboutsia* | 0.09 | 0.26 | 1.47 | 1.29 | 1.41 | 1.17 |
| *Pseudoflavonifractor* | 0.097 | 0.26 | 1.15 | 1.35 | 1.05 | 1.29 |
| *Odoribacter* | 0.099 | 0.26 | 0.094 | 0.064 | 0.11 | 0.05 |
