# Supplementary material for: Dietary supplementation with yeast hydrolysate in pregnancy influences colostrum yield and gut microbiota of sows and piglets after birth
Source: PLoS One. 2018 May 24;13(5):e0197586. doi: 10.1371/journal.pone.0197586 (PMC5967808; doi:10.1371/journal.pone.0197586)
Supplement: S5 Table — Values are presented in normalized square root transformed abundance. P values are based on the results from the Mann-Whitney test. (DOCX) [file pone.0197586.s010.docx]

| Taxa (genus) | *P* | FDR | CON mean | YD mean | CON median | YD median |
| --- | --- | --- | --- | --- | --- | --- |
| *Romboutsia* | 0.15 | 0.3 | 2.99 | 3.47 | 3.24 | 3.54 |
| *Clostridium IV* | 0.046 | 0.12 | 2.13 | 2.56 | 1.98 | 2.43 |
| *Clostridium sensu stricto* | 0.19 | 0.31 | 1.99 | 2.41 | 2.17 | 2.36 |
| *Sporobacter* | 0.0029 | 0.014 | 2.63 | 2.29 | 2.56 | 2.31 |
| *Oscillibacter* | 4.6E-06 | 0.000092 | 3.17 | 2.17 | 3.07 | 1.98 |
| *Barnesiella* | 0.2 | 0.31 | 2.03 | 1.8 | 1.88 | 1.79 |
| *Flavonifractor* | 0.000045 | 0.00045 | 2.1 | 1.67 | 2.13 | 1.65 |
| *Lactobacillus* | 0.48 | 0.56 | 1.63 | 1.59 | 1.63 | 1.36 |
| *Anaerovorax* | 0.47 | 0.56 | 1.69 | 1.57 | 1.64 | 1.52 |
| *Prevotella* | 0.42 | 0.56 | 1.21 | 1.52 | 1.12 | 1.24 |
| *Christensenella* | 0.71 | 0.75 | 1.4 | 1.44 | 1.46 | 1.54 |
| *Acetanaerobacterium* | 0.00023 | 0.0015 | 1.88 | 1.34 | 1.88 | 1.32 |
| *Holdemania* | 0.018 | 0.051 | 0.97 | 1.31 | 0.84 | 1.19 |
| *Subdoligranulum* | 0.24 | 0.34 | 1.54 | 1.26 | 1.42 | 1.14 |
| *Clostridium XlVa* | 0.69 | 0.75 | 1.24 | 1.25 | 1.21 | 1.25 |
| *Escherichia/Shigella* | 0.18 | 0.31 | 0.82 | 1.23 | 0.61 | 0.74 |
| *Eubacterium* | 0.011 | 0.037 | 0.86 | 1.2 | 0.6 | 1.08 |
| *Intestinimonas* | 0.81 | 0.81 | 1.17 | 1.2 | 1.15 | 1.2 |
| *Phascolarctobacterium* | 0.011 | 0.037 | 0.93 | 1.18 | 0.9 | 1.16 |
| *Pseudoflavonifractor* | 0.057 | 0.13 | 1.31 | 1.06 | 1.21 | 0.86 |
